# Development of a Surrogate Neutralization Assay for Norovirus Vaccine Evaluation at the Cellular Level

**DOI:** 10.3390/v10010027

**Published:** 2018-01-05

**Authors:** Xiaoli Wang, Shuxia Wang, Chao Zhang, Yu Zhou, Pei Xiong, Qingwei Liu, Zhong Huang

**Affiliations:** 1 Unit of Vaccinology and Antiviral Strategies, CAS Key Laboratory of Molecular Virology and Immunology, Institut Pasteur of Shanghai, Chinese Academy of Sciences, Shanghai 200031, China; xlwang@ips.ac.cn (X.W.); sxwang@ips.ac.cn (S.W.); chaozhang@ips.ac.cn (C.Z.); yzhou@ips.ac.cn (Y.Z.); pxiong@ips.ac.cn (P.X.); 2 University of Chinese Academy of Sciences, Beijing 100049, China

**Keywords:** norovirus, histo-blood group antigen, α1,2-fucosyltransferase 2, vaccine evaluation model, cellular level

## Abstract

Noroviruses (NoVs) are the main pathogens responsible for sporadic and epidemic nonbacterial gastroenteritis, causing an estimated 219,000 deaths annually worldwide. There is no commercially available vaccine for NoVs, due partly to the difficulty in establishing NoV cell culture models. The histo-blood group antigen (HBGA) blocking assay is used extensively to assess the protective potential of candidate vaccine-elicited antibodies, but there is still no widely used cellular evaluation model. In this study, we have established a cell line-based NoV vaccine evaluation model through the construction of human α1,2-fucosyltransferase 2-overexpressing 293T (293T-FUT2) cell lines. The 293T-FUT2 cells stably expressed H type 2 and Lewis y antigens. Virus-like particles (VLPs) of the NoV prototype strain genogroup I.1 (GI.1) and the predominant strains GII.4 and GII.17 could attach to the cell line efficiently in a dose-dependent manner. Importantly, antisera against these NoV VLPs could inhibit the attachment of the VLPs, where the inhibitory effects measured by the attachment inhibition assay correlated significantly with the antibody levels determined by the HBGA blocking assay. Collectively, our attachment inhibition assay could serve as a surrogate neutralization assay for the evaluation of NoV vaccines at the cellular level.

## 1. Introduction

Noroviruses (NoVs), belonging to the *Caliciviridae* family, are the main causes of sporadic and epidemic nonbacterial gastroenteritis [1]. It is estimated that NoVs are responsible for 219,000 deaths annually worldwide, and result in $64.5 billion in direct medical costs and indirect societal costs [2]. NoVs have been classified into seven genogroups (GI–GVII) [3], among which GI and GII are responsible for most of the human infections [4]. Although vaccines should be an effective means for preventing NoV infection, the exploration of this option, including vaccine development and evaluation, has been hindered, owing to the difficulty in establishing an applicable NoV cell culture model [5,6]. 

Several evaluation models, including the histo-blood group antigen (HBGA) blocking assay [7,8], hemagglutination inhibition (HAI) assay [9], and stem cell-derived human intestinal enteroid (HIE)-based neutralization assay [6], are currently used to assess NoV vaccines in vitro. The HIE-based neutralization assay is the only cellular model used in NoV vaccine assessment [6], but this model has not been widely used owing to the difficulty in obtaining stem cells from human intestinal tissues and the complex procedures involved in culturing HIEs. Thus, there is still a need for a new succinct model for NoV vaccine assessment at the cellular level.

The HBGA blocking assay has been used extensively to assess the protective potential of NoV vaccine candidate-elicited antibodies. HBGAs, including ABO(H), secretor, and Lewis antigens, are carbohydrates present on red blood cells and mucosal epithelial cells, or as free antigens in biological secretions [10], and that serve as attachment factors for NoVs [7,11,12]. The biosynthesis of HBGAs from disaccharide precursors (types 1–5 precursors) involves a set of glycosyltransferases, among which α1,2-fucosyltransferases (FUTs) catalyze the addition of fucose in α1,2-linkage to the precursor to form H antigens [13]. FUT1 and FUT2 are the enzymes involved in this HBGA-biosynthesizing process in humans [13]. FUT1 is solely responsible for H antigen synthesis on red blood cells [14], whereas FUT2 accounts for the secretor phenotype characterized by the presence of ABH antigens in saliva and on various epithelial cell types [13]. Individuals of the secretor phenotype are highly susceptible to NoV infection [15,16,17,18]. FUT2 shows marked preference for types 1, 3, and 4 precursors [19], and the transient expression of FUT2 in cells also results in the expression of H type 2 (H2) antigen [11,20,21,22] and NoV attachment [11,21,23]. However, no stable FUT2-overexpressing cell line is available so far.

In this study, we constructed a FUT2-overexpressing cell line-based surrogate neutralization assay for NoV vaccine evaluation. This cellular model was validated by NoV vaccine evaluation against prototype and dominant strains. Our cellular model provides a new succinct method for NoV vaccine evaluation and should greatly facilitate the development of NoV vaccines.

## 2. Materials and Methods

### 2.1. Cells and Recombinant Norovirus Virus-Like Particles

Human embryonic kidney 293T cells (CRL-3216; American Type Culture Collection (ATCC), Manassas, VA, USA) and human epithelial colorectal adenocarcinoma Caco-2 cells (HTB-37; ATCC) were cultured in Dulbecco’s modified Eagle’s medium (DMEM; Gibco, Grand Island, NY, USA) with 10% fetal bovine serum (FBS), 100 U/mL penicillin, and 100 μg/mL streptomycin at 37 °C with 5% CO_2_ [24]. The NoV GI.1 (GenBank accession no. NP_056821.2) and GII.17 (GenBank accession no. AKB94545.1) virus-like particles (VLPs) were expressed and purified using the same protocols, as used previously for GII.4 VLPs [25].

### 2.2. Antibodies 

Monoclonal antibodies against the H1, H2, Lewis b (Le^b^), and Lewis y (Le^y^) antigens were purchased from Abcam (Cambridge, Cambridgeshire, UK). Monoclonal antibodies that are specific to the A and B antigens were purchased from Thermo Fisher (Rockford, IL, USA). The β-actin-specific monoclonal antibody was purchased from Sigma (St. Louis, MO, USA). Mouse antisera against enterovirus 71 (EV71) VLP was prepared, as described previously [25]. Mouse and rabbit antisera against the GI.1, GII.4, and GII.17 VLPs (VP1) were generated in-house by immunizing mice and rabbits with the corresponding VLPs.

### 2.3. Plasmid Construction 

Genomic DNA was extracted from Caco-2 cells using a TIANamp Genomic DNA kit (Tiangen Biotech, Beijing, China) according to the manufacturer’s manual. The human *FUT2* gene was amplified from the genomic DNA with primers (forward: 5′-CCGGAATTCATGCTGGTCGTTCAGATGCC-3′; reverse: 5′-CTAGTCTAGATTAGTGCTTGAGTAAGGGGG-3′) and cloned into the *Eco*RI/*Xba*I-digested pLVX-IRES-Puro vector to create pLVX-FUT2.

### 2.4. Lentivirus Production and Transduction

To generate lentiviruses for *FUT2* overexpression, 293T cells were co-transfected with pLVX-FUT2 and two packaging vectors (psPAX2 and pMD2.G) using Lipofectamine (Invitrogen, Carlsbad, CA, USA). At 72 h post transfection, the medium containing the lentivirus particles was harvested, filtered, and transduced into 293T cells. At 48 h post transduction, the cells were treated with 1 μg/mL puromycin (Gibco) in FBS (10%)-containing DMEM for two weeks and the medium was changed on every two days. The resultant mixed-cell population was seeded into 96-well plates at one cell/well (obtained by limiting dilution) and cultured for 10 days in the presence of puromycin. Then, the cell lines derived from the single cell were picked and extended for 15 days. 

### 2.5. RNA Extraction and Quantitative RT-PCR 

Total RNAs were extracted from the wild-type 293T and *FUT2*-expressing 293T (293T-FUT2) cells using TRIzol reagent (Invitrogen) according to the manufacturer’s manual. Then, 1 μg of the isolated RNA was reverse transcribed using the PrimeScript RT reagent kit (Takara, Kusatsa, Shiga, Japan). The resultant complementary DNA (cDNA) was subjected to quantitative real-time polymerase chain reaction (qRT-PCR) analysis using *FUT2* primers (forward: 5′-ATTGGGACGTTCGGGATCTG-3′; reverse, 5′-GTCGGGGAGGGTGTAATTGG-3′) and human β-actin primers (forward: 5′-GGACTTCGAGCAAGAGATGG-3′; reverse: 5′-AGCACTGTGTTGGCGTACAG-3′), respectively. Human β-actin was used as an internal control. Data analysis was performed using the 2^−ΔΔCT^ method, as described previously [26].

### 2.6. Attachment Assay

The 293T-FUT2 and 293T cells were seeded into poly-l-Lys-coated 24-well plates (3 × 10^5^ cells per well) at one day before use. Then, 0.5 μg of the various NoV VLPs in FBS (2%)-containing DMEM was added to the cells and the plates were incubated for 1 h at 4 °C. After three washes with cold phosphate buffered saline (PBS), the cells were subjected to immunofluorescence analysis with mouse antisera against the NoV VLPs and to Western blot analysis with both mouse antisera against the NoV VLPs and monoclonal antibody against β-actin (1:1000). 

293T-FUT2 cells were seeded into poly-l-Lys-coated 96-well plates (6 × 10^4^ cells per well) at one day before use. Various doses of the NoV VLPs were added to the cells and the plates were incubated for 1 h at 4 °C. After three washes with cold PBS, the cells were subjected to cellular enzyme-linked immunosorbent assay (ELISA) with rabbit antisera against the NoV VLPs (1:1000).

### 2.7. Animal Immunization

For mouse immunization, antigens were absorbed to the Alhydrogel adjuvant (InvivoGen, San Diego, CA, USA), according to the manufacturer’s instructions. Groups of six female BALB/c mice (6–8 weeks old) were injected intraperitoneally with the antigen/adjuvant mixtures containing PBS and 10 μg of the GI.1, GII.4, or GII.17 VLP, respectively, at weeks 0 and 2. Blood samples were collected at week 4 for antibody response measurement. 

For rabbit immunization, adult New Zealand White rabbits were injected subcutaneously with 150 μg NoV VLPs plus complete Freund’s adjuvant (Sigma) on day one. Then, the rabbits were given a booster injection with 150 μg NoV VLPs plus incomplete Freund’s adjuvant (Sigma) on days 21 and 42, respectively. At two weeks after the last booster, the rabbits were scarified, and sera were collected.

All the animal studies were approved by the Institutional Animal Care and Use Committee at the Institut Pasteur of Shanghai (Permit Number: A2016013).

### 2.8. Histo-Blood Group Antigen Blocking Assay

The HBGA blocking assay was performed, as described previously [25], with a minor modification, in that the VLP concentration was 1 μg/mL. The 50% blocking titer (BT50) was defined as the highest serum dilution that blocks at least 50% of VLP binding relative to the level determined in the absence of antibody pretreatment, as described previously [25,27,28].

### 2.9. Attachment Inhibition Assay 

The 293T-FUT2 cells were seeded in poly-l-Lys-coated 96-well plates (6 × 10^4^ cells per well) at one day before use. A 50-µL aliquot of the two-fold serial dilution of each serum sample was mixed with an equal volume of the various NoV VLPs (1 μg/mL) and incubated for 1 h at 37 °C. The resultant VLP/serum mixtures were then added to the 293T-FUT2 cells and the plates were incubated for 1 h at 4 °C. After three washes with cold PBS, the cells were subjected to cellular ELISA using rabbit antisera against the NoV VLPs (1:1000), as described in Section 2.11. The inhibitory effect of antisera was denoted as neutralization titer, because attachment inhibition assay simulated pre-attachment neutralization assay. The 50% neutralization titer (NT50) was defined as the highest serum dilution that inhibits at least 50% of VLP attachment relative to the level determined in the absence of antibody pretreatment.

### 2.10. Immunofluorescence Assay 

The immunofluorescence assay was performed, as described previously, with minor modifications [29]. In brief, after being fixed with 4% paraformaldehyde and blocked with NETG buffer (150 mM NaCl, 5 mM EDTA, 50 mM Tris-HCl, 0.05% Triton X-100, and 0.25% gelatin) [30], the cells were incubated with antibodies specific to the various NoV VLPs (1:1000) or HBGAs (1:100) diluted in NETG buffer at 4 °C overnight. Subsequently, the cells were incubated with Alexa-488-conjugated secondary antibody and 4′,6-diamidino-2-phenylindole (DAPI) in NETG buffer. The resultant cells were analyzed by confocal microscopy (Olympus FV-1200, Olympus, Tokyo, Japan).

### 2.11. Cellular Enzyme-Linked Immunosorbent Assay 

Cells were fixed with 4% paraformaldehyde for 30 min at room temperature and then blocked with blocking buffer (containing 10% FBS and 10% bovine serum albumin in PBS) for 1 h at 37 °C. Then, the cells were incubated with antibodies specific to the various NoV VLPs (1:1000) or HBGAs (1:100) for 1–2 h at 37 °C. This was followed by incubation with the appropriate horseradish peroxidase-conjugated secondary antibody. Measurement of the optical density at 450 nm (OD_450nm_) was performed, as described previously [31].

### 2.12. Statistical Analysis

Statistical significance was calculated by the Student’s *t*-test or Spearman’s correlation using GraphPad Prism version 5, and are indicated as follows in the figures: n.s. *p* ≥ 0.05; * *p* < 0.05; ** *p* < 0.01; and *** *p* < 0.001.

## 3. Results

### 3.1. Establishment of Stable FUT2-Overexpressing 293T Cell Lines

To establish stable *FUT2*-overexpressing cell lines, plasmid pLVX-FUT2 containing the human *FUT2* gene was constructed and transfected into 293T cells together with packing vectors to generate lentivirus particles. The 293T cells were then transduced by the lentivirus particles and screened with puromycin, to yield a mixed-cell population. Subsequently, eight cell lines (#1–8) were obtained from the mixed-cell population by the limiting dilution method. 

**Figure 1:**
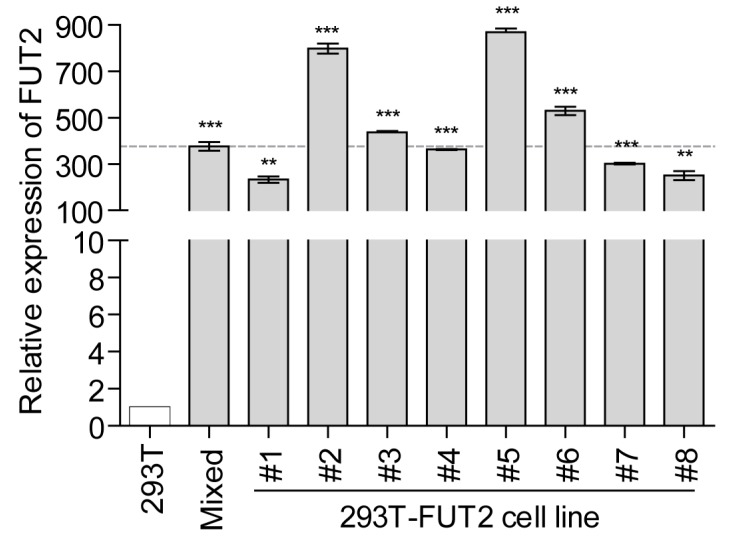
Expression of α1,2-fucosyltransferase 2 (*FUT2*) in 293T-FUT2 cells. Total RNAs from cells of wild-type 293T, mixed-cell population, and 293T-FUT2 cell lines were extracted and analyzed for *FUT2* expression. The expression in wild-type 293T cells was set to 1 and used as the calibrator. Data are the means ± standard deviation (SD) of triplicate experiments. Mixed, mixed-cell population. Statistical significance was determined by the Student’s *t*-test using GraphPad Prism version 5, and values are indicated as follows: ** *p* < 0.01; *** *p* < 0.001.

To validate the expression of *FUT2* in 293T-FUT2 cells, total RNAs were extracted and subjected to quantitative qRT-PCR. When compared with the *FUT2* RNA level in wild-type 293T cells, the level was significantly increased by 100–900 folds in the #1–8 cell lines and mixed-cell population of 293T-FUT2 (Figure 1). Furthermore, the mRNA expression of *FUT2* in cell lines #2, #3, #5, and #6 was slightly higher than that in the mixed-cell population (Figure 1). The expression of FUT2 protein was not determined by Western blot assay owing to the very low sensitivity of the FUT2-specific antibody (data not shown).

### 3.2. Expression of HBGAs in 293T-FUT2 Cells

**Figure 2:**
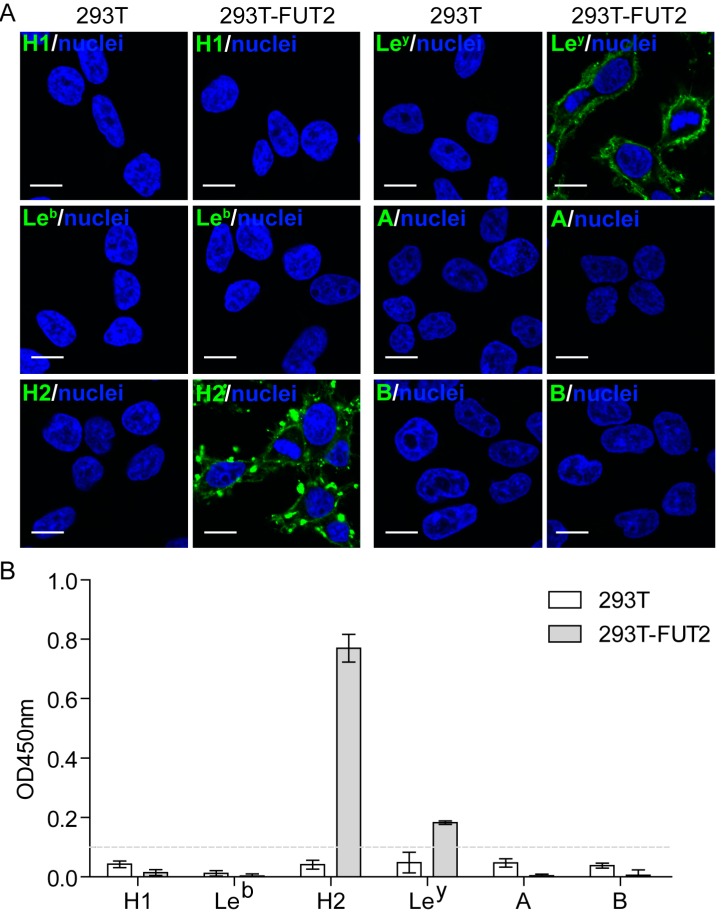
Expression of histo-blood group antigens (HBGAs) in 293T-FUT2 cells. Wild-type 293T cells and transduced 293T-FUT2 cells were examined for their HBGA expression through immunofluorescence analysis and cellular enzyme-linked immunosorbent assay (ELISA) using monoclonal antibodies specific to each of six types of HBGAs (H1, Le^b^, H2, Le^y^, A, and B) as described in Materials and Methods. (**A**) Immunofluorescence analysis of HBGA expression in 293T-FUT2 cells. Scale bar, 10 μm. (**B**) Cellular ELISA analysis of HBGA expression in 293T-FUT2 cells. Data are the means ± SD of the optical density at 450 nm (OD_450nm_) values subtracted from the blank value (cells without incubation with primary antibody) for quadruple wells. The dash line indicates the cutoff, which is defined as 0.1 OD unit above the blank. All of the experiments were replicated three times.

Catalyzed by FUT2, a fucose could be added to the precursors of HBGAs in α1,2-linkage to generate the H type antigens, including H1, H2, H3, and H4, which are further used as precursors to synthesize Le^b^, Le^y^, A, or B antigens [13]. To investigate the cellular expression of HBGAs, the 293T and 293T-FUT2 cells were fixed and analyzed by immunofluorescence and cellular ELISA. As shown in Figure 2, no positive signal was detected in 293T cells by either the immunofluorescence assay or cellular ELISA assay, suggesting the absence of HBGAs in the 293T cells. In contrast, the 293T-FUT2 cells exhibited significant reactivity with H2 and Le^y^ antigen-specific antibodies, but not with H1, Le^b^, A, or B antigen-specific antibodies (Figure 2A). Consistently, only antibodies against H2 or Le^y^ could react with 293T-FUT2 in the cellular ELISA analysis (Figure 2B). These results demonstrate that both H2 and Le^y^ antigens are expressed in the 293T-FUT2 cells.

### 3.3. Attachment of Norovirus Virus-Like Particles to 293T-FUT2 Cells

**Figure 3:**
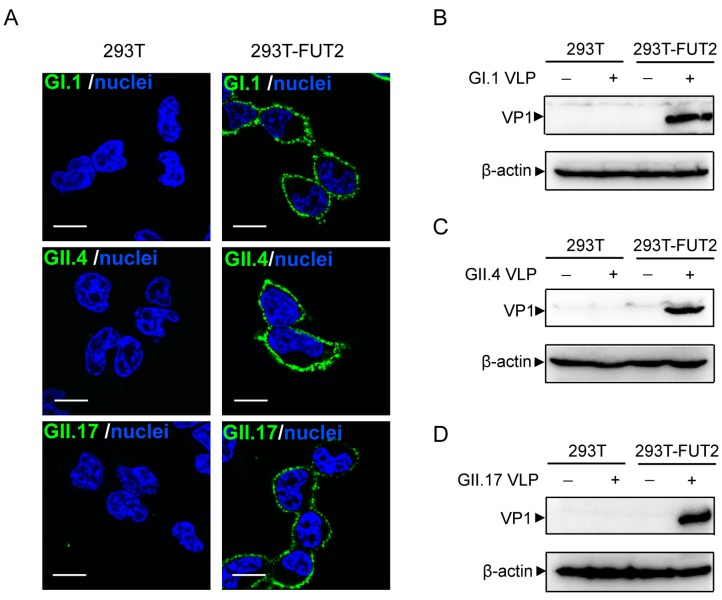
Binding of norovirus virus-like particles (NoVs VLPs) to 293T-FUT2 cells. NoV VLPs were incubated with 293T and 293T-FUT2 cells for 1 h at 4 °C and subjected to immunofluorescence and Western blot analyses with mouse antisera against GI.1, GII.4, or GII.17 VLPs. β-Actin was chosen as an internal control. (**A**) Immunofluorescence analysis of NoV VLPs binding to 293T-FUT2 cells. Scale bar, 10 μm. (**B**–**D**) Western blot analysis of NoV VLPs binding to 293T-FUT2 cells. All of the experiments were replicated three times.

Previous studies have shown that NoVs bind to HBGAs in a strain-dependent manner [32,33]. The GI.1 VLP bound to H1, H2, H3, Le^b^, and Le^y^ antigens [34,35], whereas the GII.4 (VA387) VLP interacted with H1, H3, Le^b^, and Le^y^ antigens [34], and GII.17 had a wide binding spectrum to saliva samples [36]. To explore whether NoV VLPs could attach to the 293T-FUT2 cells, GI.1, GII.4, or GII.17 VLPs were incubated with the cells and detected with VLP-specific antisera. As shown in Figure 3A, no signal was detected in 293T cells, suggesting that these three VLPs do not attach to these wild-type cells. In contrast, significant signals were observed on the periphery of 293T-FUT2 cells (Figure 3A), indicating that NoV GI.1, GII.4, and GII.17 VLPs could bind efficiently to the *FUT2*-expressing 293T cells. Similarly, Western blot analysis showed obvious bands only when the NoV VLPs were incubated with 293T-FUT2 cells (Figure 3B–D). These results indicated that NoV VLPs of both prototype and epidemic strains could efficiently attach to our constructed 293T-FUT2 cells.

### 3.4. Establishment of a Cellular ELISA Assay to Detect Norovirus Virus-Like Particles Attachment 

**Figure 4:**
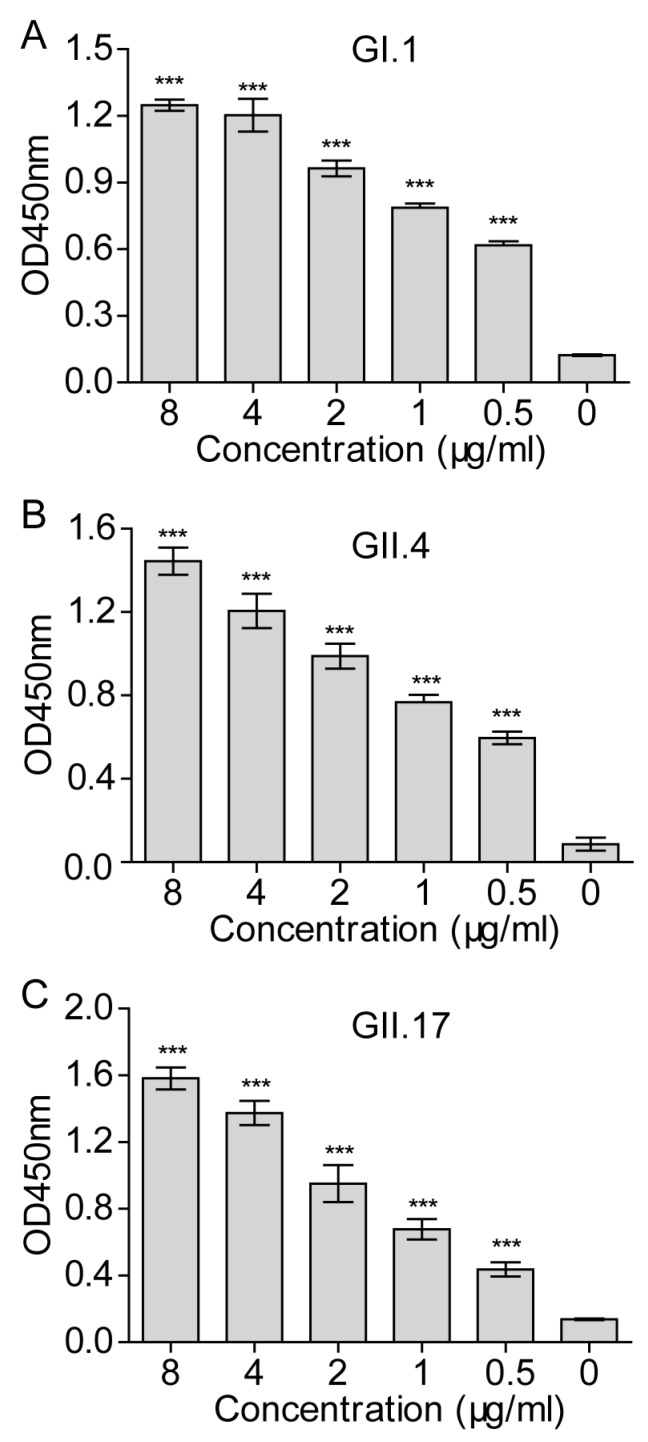
Norovirus virus-like particles (NoVs VLPs) attachment to 293T-FUT2 cells in a dose-dependent manner. Various doses of NoV VLPs were incubated with 293T-FUT2 cells for 1 h at 4 °C and detected by cellular ELISA with rabbit antisera against GI.1, GII.4, or GII.17 VLPs. (**A**) GI.1VLP attachment to 293T-FUT2 cells; (**B**) GII.4 VLP attachment to 293T-FUT2 cells; (**C**) GII.17 VLP attachment to 293T-FUT2 cells. Data are the means ± SD of OD_450nm_ values for triple wells. Statistical significance was determined by the Student’s *t*-test using GraphPad Prism version 5, and values are indicated as *** *p* < 0.001. All of the experiments were replicated three times.

In order to measure NoV VLP attachment simply and quantitatively, a cellular ELISA was developed. The 293T-FUT2 #6 cell line was randomly chosen to ensure consistent performance of the cellular ELISA. To verify this assay, various doses of each NoV VLP were incubated with 293T-FUT2 cells and detected with the VLP-specific antiserum. As shown in Figure 4A, the GI.1 VLPs bonded to 293T-FUT2 cells efficiently in a dose-dependent manner, and a similar tendency was observed with the GII.4 (Figure 4B) and GII.17 (Figure 4C) VLPs. These results indicate that the cellular ELISA could serve as a simple method for determining NoV VLP attachment.

### 3.5. Inhibition of the NoV VLP Attachment to 293T-FUT2 Cells by NoV VLP-Specific Antisera

**Figure 5:**
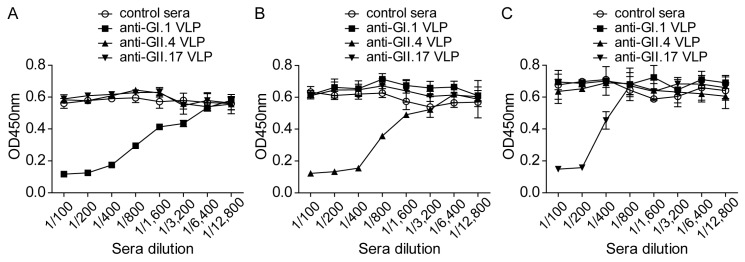
Inhibition of the norovirus virus-like particle (NoV VLP) attachment to 293T-FUT2 cells. NoV VLPs were preincubated with serially diluted mouse antisera for 1 h at 37 °C, and the resulting VLP/antisera mixtures were added to 293T-FUT2 cells. The attached VLPs were detected with VLP-specific rabbit antisera. (**A**) GI.1 VLP-specific antisera inhibited GI.1 VLP attachment to 293T-FUT2 cells; (**B**) GII.4 VLP-specific antisera inhibited GII.4 VLP attachment to 293T-FUT2 cells; (**C**) GII.17 VLP-specific antisera inhibited GII.17 VLP attachment to 293T-FUT2 cells. The enterovirus 71 (EV71) VLP-specific antisera were used as negative control sera. Data are the means ± SD of OD_450nm_ values for triple wells. All of the experiments were replicated three times.

Given that NoV VLPs could bind to 293T-FUT2 cells efficiently in a dose-dependent manner (Figure 4), a cellular ELISA-based attachment inhibition assay was established to explore whether NoV VLP-specific mouse antisera could block the VLP attachment. As shown in Figure 5, antisera against GI.1 (Figure 5A), GII.4 (Figure 5B), or GII.17 (Figure 5C) VLPs revealed similar inhibitory trends in a dose-dependent manner against binding of the corresponding VLP to 293T-FUT2 cells, whereas control serum did not inhibit the attachment of the three VLPs. Moreover, the three antisera against NoV VLPs did not show any inhibitory effect against heterogeneous VLPs (Figure 5A–C). These results suggested that the inhibitory effect conferred by NoV VLP-specific antisera was specific and the cellular ELISA-based attachment inhibition assay may be used for the evaluation of NoV vaccines.

### 3.6. Evaluation of Norovirus Vaccine In Vitro at the Cellular Level

**Figure 6:**
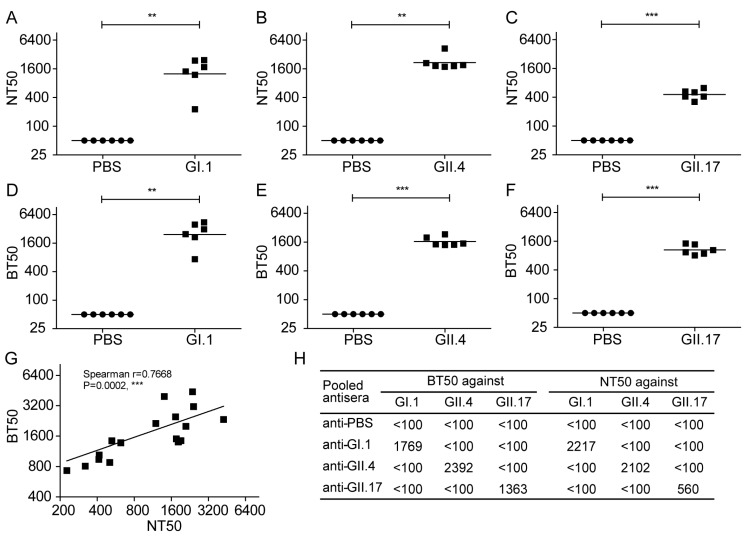
Evaluation of norovirus vaccines by a cellular ELISA-based attachment inhibition assay. Four groups of mice were immunized intraperitoneally with GI.1 virus-like particles (VLPs), GII.4 VLPs, GII.17 VLPs, or PBS, respectively, at weeks 0 and 2. Sera were collected from all immunized mice at 2 weeks after the last immunization and subjected to attachment inhibition assay and histo-blood group antigen blocking assay. (**A**) The 50% neutralization titers (NT50s) and (**D**) 50% blocking titers (BT50s) of sera from the GI.1 VLP group against GI.1; (**B**) NT50s; and, (**E**) BT50s of sera from the GII.4 VLP group against GII.4; (**C**) NT50s; and, (**F**) BT50s of sera from the GII.17 VLP group against GII.17. Samples that showed less than 50% blockade or inhibition at a 1:100 dilution (the lowest dilution tested) were assigned a titer of 50 for geometric mean titer computation. Each symbol represents one mouse, and the line indicates the geometric mean value of the group. Statistical significance was determined by the Student’s *t-*test using GraphPad Prism version 5, and values are indicated as follows: ** *p* < 0.01; *** *p* < 0.001; (**G**) Spearman’s correlation between NT50s and BT50s. Each square represents one mouse. Spearman’s *r* and *p* values are shown; (**H**) Potential neutralization of the pooled antisera against GI.1, GII.4 and GII.17. The lowest dilution tested is 1:100. All of the experiments were replicated three times.

To compare this attachment inhibition assay with the classical HBGA blocking assay, four groups of mice were immunized twice with the GI.1, GII.4, or GII.17 VLPs or PBS, and the collected sera were used to determine the antibody responses using the two assays. As shown in Figure 6, the PBS group did not show any inhibitory or blocking effects against the NoVs, even at 1:100 dilution (the lowest dilution tested), as measured by either the attachment inhibition assay or HBGA blocking assay. However, sera from the GI.1 VLP-immunized group showed efficient inhibitory effect against GI.1 in both the attachment inhibition assay (Figure 6A) and HBGA blocking assay (Figure 6D), with geometric mean titers (GMTs) of 1250 and 2435, respectively. Furthermore, sera from the GII.4 VLP-immunized group displayed comparable potential against GII.4 VLPs in the attachment inhibition assay (GMT = 2156) (Figure 6B) and HBGA blocking assay (GMT = 1644) (Figure 6E). Sera from the GII.17 VLP-immunized group also exhibited a prohibitory effect at the attachment step, with a GMT of 457 (Figure 6C), which was slightly lower than that of the BT50 value (GMT = 1054) (Figure 6F). As expected, a significant correlation (Spearman’s *r* = 0.7668, *p* = 0.0002) was observed between these two assays (Figure 6G). In addition, the cross-neutralization potential of pooled antisera was examined using the three VLPs. As shown in Figure 6H, pooled antisera form GI.1, GII.4, or GII.17 groups showed inhibitory effects against only homogeneous VLPs, but not heterogeneous VLPs, using the attachment inhibition assay. This was in line with the result measured by HBGA blocking assay, demonstrating that the attachment inhibition assay could be used to evaluate cross-neutralization potential of NoV vaccine. Collectively, the attachment inhibition assay could be used as a surrogate neutralization assay for NoV vaccine assessment.

## 4. Discussion

The routine cellular models available for NoV vaccine evaluation are limited. In our study, we have generated a *FUT2*-overexpressing cell line for the first time and established an attachment inhibition assay based on this cell line for NoV vaccine evaluation. We demonstrated that *FUT2* overexpression in 293T cells led to H2 and Le^y^ antigen expression and NoV VLP attachment, which could be inhibited by specific antisera in a dose-dependent manner. Moreover, we applied the attachment inhibition assay in vaccine evaluation against NoV prototype (GI.1) and predominant strains (GII.4 and GII.17) [4,37,38,39,40,41], and showed significant correlation of this assay with the classical HBGA blocking assay. 

H2 and H3 antigens were expressed in 293T cells with transient FUT2 expression [22], whereas H2 and Le^y^ antigens were detected in 293T-FUT2 cells. Whether H3 antigens are expressed in 293T-FUT2 cells was not determined, owing to the lack of an H3-specific antibody. However, the H1, Le^b^, A, and B antigens were not detected in the 293T-FUT2 cells. The absence of A and B antigens was likely caused by a lack of A and B enzymes that catalyze the formation of these two antigens from H antigens, respectively [13]. The H1 antigen is used as a precursor in Le^b^ synthesis, and thus the lack of H1 and Le^b^ antigens might be caused by type 1 precursor deficiency, which has been reported in the Chinese hamster ovary carcinoma cell line [11]. 

Caco-2 cells exist in two forms: undifferentiated and differentiated. Undifferentiated Caco-2 cells heterogeneously express H1, H2, and Le^b^ antigens [30], and NoV VLP attached to only a certain population of cells [30,42]. Undifferentiated Caco-2 cells could spontaneously differentiate into polarized columnar cells with the characteristics of small intestine after reaching confluency [43]. Although, differentiated Caco-2 showed increased binding activity to NoV VLP [30,44], NoV VLP could be detected only on certain cells or clusters of the cell layer [30,45] and the HBGA expression of differentiated Caco-2 reportedly differed [30,45,46]. Thus, Caco-2 cells were not used in NoV vaccine evaluation. Unlike Caco-2 cells, 293T-FUT2 cells stably expressed H2 and Ley antigens, and NoV VLP could attach to the cells homogeneously. Furthermore, NoV GI.2, GI.3, GI.7, GII.3, GII.6, GII.7, and GII.10 reportedly bound to H2, H3, and/or Le^y^ antigens [47,48,49,50], it would be reasonable to presume that 293T-FUT2 cell-based evaluation model could be used to evaluate NoV vaccine against these strains. 

Although, cellular ELISA-based attachment inhibition assay may be not as sensitive as HIE-based neutralization assay [6], it has the following distinct advantages over the existing NoV vaccine evaluation models. First, cell membrane-bound HBGAs might be more restricted than free HBGAs owing to the orientational constraints that are caused by their lateral interactions with other membrane lipids and proteins [51]; therefore, the state of HBGAs on 293T-FUT2 cells is more likely to simulate that of the host cell rather than free HBGAs. Moreover, secretor products (H2 and Le^y^ antigens) expressed in 293T-FUT2 cells are also efficiently expressed in human intestinal organoids representing the individual secretor, and function as attachment receptors in NoV infection [52]. Thus, our cellular model may more authentically exhibit neutralizing activity against NoVs. Second, our vaccine evaluation model is more consistent, as this *FUT2*-overexpressing cell line can stably express HBGAs. Moreover, the consistency of the HBGA blocking assay is highly affected by the HBGA lot [53], whereas the HAI assay could be influenced by the source of human erythrocytes [54], and the B-cell culture model [5] of NoVs is easily influenced by the FBS source and virus stock [55]. Third, the cell line used in the evaluation model is more readily obtained when compared with the HIEs [6] or human erythrocytes used in HAI assays [9]. Finally, this evaluation method is simple to operate, and could also be used for high-throughput screening of antibodies and drugs against NoVs.

In conclusion, the cellular ELISA-based attachment inhibition assay established in this study could be used as a surrogate neutralization assay for NoV vaccine evaluation. The *FUT2*-ovexpressing cells can also be useful for studying NoV-host cell interactions.
